# O048. Chronic migraine, cluster headache or both? The suggestion of botulinum toxin

**DOI:** 10.1186/1129-2377-16-S1-A107

**Published:** 2015-09-28

**Authors:** Patrizia Ripa, Anna Ambrosini, Antonio Carolei, Simona Sacco

**Affiliations:** Institute of Neurology, Department of Applied Clinical Sciences and Biotechnology, University of L'Aquila, L'Aquila, Italy; Headache Unit, IRCCS Neuromed, Pozzilli, (IS) Italy

## Introduction

Cluster headache (CH) is an uncommon primary headache that may be difficult to diagnose when comorbid with other headache disorders[1]. We describe two patients with a challenging diagnosis, emerging after treatment with botulinum toxin (BTX).

## Case 1

A 58-year-old man presented with a three decades history of almost daily unilateral, not side-locked, pulsating head pain with severe exacerbations, lasting 4-72 hours, associated with photo-, phono-, and osmophobia, conjunctival injection, increasing with physical activity. Indomethacin overuse was present and the patient met diagnostic criteria for both chronic migraine (CM) and medication-overuse headache. He had tried, over the years, several drugs (antiepileptics, such as, valproate and topiramate, antidepressants, calcium-channel blockers, and beta blockers) and non-pharmacological treatments (biofeedback, acupuncture) with only transient improvements. Treatment with BTX, according to the PREEMPT injection protocol[2], was started together with venlafaxine and steroids for two weeks with mild improvement of the headache. After two cycles of treatment with BTX, the patient developed attacks of stabbing pain localized behind his right eye, spreading to the right side of the head, with marked conjunctival injection. The pain recurred 8-10 times per day, usually lasting about 60 minutes, and was associated with restlessness. CH was diagnosed. Steroids and verapamil, as well as subcutaneous sumatriptan in acute attacks, were started with cessation of the CH attacks and persistence of migraine attacks. Four months later, CH attacks relapsed and were again successfully treated.

## Case 2

A 41-year-old woman presented with a history of pulsating pain in the left, sometimes right, temporal region and behind the eye associated with ipsilateral lacrimation, photo-, phonofobia and vomiting occurring 15 times a month, lasting 12-24 hours and fulfilling diagnostic criteria for CM. Drug prophylaxis with amitriptyline and flunarizine was initiated but stopped for ineffectiveness and side effects. Treatment with BTX, according to the PREEMPT[2] injection protocol was started with complete cessation of attacks. After three cycles of treatment, the patient developed stabbing pain localized behind her left eye with autonomic signs, spreading to the left side of the head, recurring 3-4 times per day and lasting 60-120 minutes, fulfilling diagnostic criteria for CH. BTX was stopped; treatment with steroids and topiramate was initiated with resolution of the attacks. Off the cluster, the patient autonomously withdrew all treatments with recurrence of migraine-type headache; BTX was restarted with good response.

## Discussion

In our opinion, in both the cases there was a comorbidity between chronic migraine and CH. In the former case the presence of CH was covered up by the prominence of migraine features and by the effects of preventive and acute phase treatments used to treat migraine that may also have limited the disclosure of CH symptoms thus making its diagnosis more challenging. In the second case, CH developed during treatment with BTX and, although unlikely, we do not know if and how BTX could have trigger CH.

Written informed consent to publication was obtained from the patient(s).
